# High school rugby coaches’ knowledge and opinions of concussion in KwaZulu Natal province in South Africa: an ecological cross-sectional study

**DOI:** 10.1186/s13102-024-00930-5

**Published:** 2024-06-24

**Authors:** Daniel Garnett, Saul Cobbing, Carel Viljoen, Jon Patricios

**Affiliations:** 1 Physiotherapy Department, College of Health Sciences, University of KwaZulu-Natal, Durban, South Africa; 2 Department of Sport, Health Sciences and Social Work, Faculty of Health and Life Sciences, Oxford Brookes University, Oxford, UK; 3 The Institute for Education Research, University Health Network, Toronto, Canada; 4 Department of Physical Therapy, University of Toronto, Toronto, Canada; 5 Department of Physiotherapy, Faculty of Health Sciences, University of Pretoria, Pretoria, South Africa; 6 Amsterdam Collaboration On Health & Safety in Sports, Department of Public and Occupational Health, Amsterdam Movement Science, Amsterdam UMC, Amsterdam, the Netherlands; 7 Wits Sport and Health (WiSH), School of Clinical Medicine, Faculty of Health Sciences, University of the Witwatersrand, Johannesburg, South Africa

**Keywords:** Rugby Union, Sports, Adolescent, Injury prevention

## Abstract

**Background:**

Concussions in Rugby Union are common with an increased risk to adolescent players. Coaches are key to injury prevention and a greater understanding of their knowledge and sentiments may guide future initiatives. There is a lack of data on rugby coaches, especially in South Africa. This study aimed to investigate the knowledge and opinions of high school rugby coaches regarding concussion management.

**Methods:**

This cross-sectional study of 37 high school rugby coaches in South Africa, was conducted via a self-reported questionnaire. Concussion knowledge was scored for correct answers only with closed-question scaling methods to measure the importance of items of concussion management using a graphical rating scale. An attitude scale (Likert) was used to assess self-reported opinions and behaviours. Associations were calculated for participant characteristics and overall concussion injury knowledge.

**Results:**

More participants showed good overall knowledge of ≥ 75% (*n* = 22, 59% vs. *n* = 15, 40%), especially those with greater coaching experience (*p* = 0.021). Player welfare was perceived more important than player performance (185 vs. 164), with concussion prevention most important (184 of 185). Appealing characteristics of an injury prevention programme were the improvement of player skill (173, SD ± 0.75, mean 4.68), being adaptable (171, ± 0.86, 4.62), and being completed in the warm-up (167, ± 0.93, 4.51). The biggest perceived barriers were duration (138, ± 1.59, mean 3.73), effort (130, ± 1.56, 3.51), compliance and lack of knowledge (both 127, ± 1.68, 3.43).

**Conclusion:**

These results support the implementation of ongoing concussion education for rugby coaches and identify areas for promoting awareness and knowledge of concussion injury prevention, identification, and specific management of younger athletes. Appealing characteristics and barriers are highlighted and may allow for improved implementation and adherence to concussion prevention programmes.

**Supplementary Information:**

The online version contains supplementary material available at 10.1186/s13102-024-00930-5.

## Key points

• The risk of concussion to female athletes and younger athletes was under-reported and should be addressed in future coach-focused education initiatives.

• Successive educational approaches may be effective in coaches’ knowledge retention, although the most appropriate delivery methods should be researched further.

• The ideal injury prevention programme should improve player skill whilst reducing the risk of injury, be adaptable, completed during the warm-up and not take much time or effort to complete.

**Supplementary Information:**

The online version contains supplementary material available at 10.1186/s13102-024-00930-5.

## Background

Concussion in Rugby Union (“rugby”) is common, with potential shorter and longer-lasting symptoms, especially in adolescents [1–3]. The longer-lasting symptoms of concussion are associated with deficits in health-related quality of life affecting aspects of social, emotional and psychological well-being [4–7]. The global population-based rate is above 600 per 100,000 people and is recognized by the WHO as an important public health problem [8]. There is a three- to four-fold higher risk of sustaining a consecutive concussion when having a history of a previous concussion [9]. The Amsterdam International Consensus Statement on Concussion in Sport (6^th^ CIS) describes a sport-related concussion as a traumatic brain injury caused by a direct blow to the head, neck, or body resulting in an impulsive force being transmitted to the brain that occurs in sports and exercise-related activities which may include signs and symptoms presenting immediately or evolving over minutes or hours [10].

In Canada, there are approximately 12 concussions for every 1000 people costing $1.5 billion, with many more indirect costs [11–15]. In South Africa, public health reporting systems are not as developed as in other countries, such as Canada [16]. However, injury reporting of injury burden and injury epidemiology at South African elite youth rugby tournaments have been comprehensively investigated with multiple factors that may contribute to concussion risk factors having been identified [1, 17–23]. The high standard of concussion management at these elite youth events is comparable to that of international adult matches [24], with attending sports medicine doctors and physiotherapists being qualified in pitch-side concussion management and return-to-play guidelines [25]. However, these elite events do not reflect the concussion management resources routinely available to the majority of the rugby-playing school community in a developing country with limited delivery of services in health and education [26]. In low socioeconomic environments, as seen in many parts of South Africa, multiple barriers exist for students to participate in sports [27, 28], and this will extend to coaches, who are most often teachers first, and sports coaches second. Organized school sports participation has reduced since 2014 [28], and due to resource limitations, unlike at the elite rugby tournaments, qualified healthcare professionals will not be on hand for most of the school sport-playing population, and injury reporting would be a distant consideration.

However, SA Rugby provides the freely accessible BokSmart injury prevention programme which includes, amongst other features, mandatory biennial training workshops for rugby coaches and referees [29]. In South Africa, New Zealand (NZ) and England, rugby coaches are identified as the preferred source of player education on safe techniques and are acknowledged as being influential for player behaviour [30–32]. A recent report from NZ showed rugby coaches scored significantly higher than players for knowledge and attitudes of concussion with coach (but not player) education, in a similar approach to BokSmart, called RugbySmart [33]. In South Africa, this coach-orientated education has been associated with positive behavioural change facilitating injury prevention in rugby players [30], although the depth of understanding of concussion knowledge attainment and retention requires further and repeated evaluation.

In other sports, the implementation of exercise-based injury prevention programmes, before or during training sessions, has been successful in reducing the number of injuries [34, 35]. Similarly, the composition of rugby training sessions has an effect on the incidence of a broad spectrum of injuries, including concussion, in both adolescent and adult populations [36, 37], with neuromuscular training (NMT) being acknowledged and recommended for concussion prevention by all expert panelists of the Concussion in Sport Group (CISG) following the findings of a systematic review on prevention of concussion [5, 24].

In most sports at school level in South Africa, coaches are responsible for the planning of training sessions, the composition of which will be influenced by the knowledge, experience, resources and opinions of these coaches [38]. Previously, in coach education initiatives like RugbySmart in NZ and HeadCase in England, coaches were found to have the lowest concussion knowledge compared to players, medical staff and referees [39–42]. A limited knowledge of the management of concussion injuries has also been described amongst cohorts of South African rugby coaches [43]. Coaches have been shown to instruct with similar methods to how they were coached as a player [44], and in some instances, coaches were willing to put pressure on concussed players to continue playing and/or on the medical team to allow a player with a concussion to continue [42]. The preferences and values of the targeted populations, like patients in medical care and in this research, coaches in coach-education, is an integral component of evidence-based practice [45, 46], and is well-placed in Stage 5 of the TRIPP framework of injury prevention to understand the context of personal, environmental, societal and sports delivery factors that may enhance or be barriers to implementation [47, 48].

A deeper understanding of the factors that guide the decisions and actions of this important stakeholder group is critical to the success of appropriate concussion management in rugby. The purpose of this study was to gain insight into the:

(i) knowledge of South African rugby coaches regarding concussion prevention,
(ii) opinions of South African rugby coaches on concussion prevention, and
(iii) barriers, concerns, or strategies to implementing a rugby injury prevention programme in South African high schools.

## Methodology

### Study design and setting

This cross-sectional design study was conducted in registered rugby-playing schools in the KwaZulu-Natal (KZN) province in the eastern coastal region of South Africa, an area with a long tradition of schoolboy rugby. Different funding models in these schools result in a wide disparity in resource allocation and availability, with some schools having world-class facilities and other schools not having grass on their sports fields [49]. The historical context of the South African political landscape may be the reason for many of the differences in resources at these schools which ultimately results in social inequality with some people being disadvantaged, relative to others [50].

### Participants

Total population sampling was used due to the small number of participants and is considered to be most appropriate as generalisations can be justified for this specific cohort [51]. Other forms of random or stratified sampling were not possible as the researcher did not have access to a full database of the members of the population, rather to one person who disseminated the questionnaire and study information. All registered coaches of the KZN Rugby Union High School database were invited to participate in this study (*n* = 126).

### Research instrument

The research instrument included a self-reported online questionnaire (Appendix A). The first draft questions were developed by the principal researcher who identified relevant topics after a review of the available literature.

The authors used previously reported guidelines and examples of closed-question scaling methods, Rating and Attitude scales, to generate a sequence of values, upon which the measured values were placed [52]. A 10–point numerical rating scale was used to rate the importance of some answers, with one being the lowest value and 10 being the highest value for example; ‘Please rate the following questions on a scale of 1–10 where 1 is the lowest value and 10 is the highest value: 2.1 How important is it to you, to prevent injury to your players?’.

To measure an individual participant’s predisposition to an item, an attitude scale is recommended [52]. Although not validated in this study, a five-point Likert scale, previously reported in research on coach perception and education [53], was used to quantify opinions with numerical values increasing with escalating support for an opinion. This method was deemed more appropriate for some questions and is more suitable for assessing participants’ experiences [54]. For these questions, scores for opinion were added and the total scores were ranked as an indication of the importance of each item. The lowest total score representing “not at all” would be 37 (*n* = 37 × 1) and the maximum score representing “very” would be 185 (*n* = 37 × 5).

Face and content validity were established after appraisal of the questionnaire by two high school coaches, a second physiotherapist, and a sports medicine physician. Recommendations, corrections, and re-ordering of answers were implemented to reduce ambiguity and repetition and to improve the overall flow of the answering process. The second draft of the questionnaire included 37 questions. This paper-based format was converted to an electronic format using Google Forms. This was deemed necessary to improve delivery as well as to reduce unnecessary contact and risk for participants during the global pandemic. The electronic form was used in a pilot study with high school coaches (*n* = 14) to identify any further weaknesses or ambiguities in the research instrument. No further recommendations were received, and the final format received only cosmetic enhancements before being used in the main study.

### Procedures

The University of KwaZulu-Natal (UKZN) Biomedical Ethics Committee approved the study (BREC 00000961/2020). Further, permission and support were obtained from provincial bodies and the Chairman of the KZN High School Rugby Association. A descriptive email with a study link was sent electronically to all registered email addresses on the High School coach database. All relevant study information was provided, and coaches were invited to participate in the study. The study link allowed access to the online questionnaire and could be completed on any web-enabled electronic device, including smartphones. Two weeks after the initial email, a follow-up email was sent to all registered email addresses to remind and encourage participation in the study. No additional questionnaires were received one month after the initial call for participants, and the data collection phase was concluded (4 weeks).

### Data analysis

Data was entered into Microsoft Excel and analyzed using Statistical Package for the Social Science (SPSS), version 28. Descriptive and inferential statistics was calculated, including the percentage and 95% confidence interval positive response to questions. Kolmogorov–Smirnov test was used to test normality of the data. Fisher's exact test was used to test the association between Coaches’ overall knowledge versus demographic variables. One-sample t-test was used to compare the mean Coaches’ opinions on prevention of injury and player performance and hypothesized value (i.e. 3). Furthermore, multi-covariate logistic regression was used to check the effects of demographic characteristic on Coaches’ overall knowledge. All the tests were two-sided and all *p*-values reported was tested at α = 0.05 level.

Participants’ scores for knowledge of concussion and injury prevention were calculated with a threshold of 75% and separated into two categories: ‘good overall knowledge’ (≥ 75%) and ‘not good overall knowledge’ (< 75%). These results were tabled according to participant background characteristics (Table 1). Participant answers that included ‘Maybe’ were considered to be incorrect for the purposes of calculation.

**Table 1:** Participant characteristics and associations between overall knowledge versus background variables

		Participant characteristics n (%)	Overall knowledge		Fisher exact *p*-value
			Not good n (%)	Good n (%)	
Age	< 40 years	27 (73)	10 (37)	17 (63)	0.436
	40–59 years	9 (24)	5 (56)	4 (44)	
	+ 60 years	1 (3)	0 (0)	1 (100)	
Rugby coaching experience	1–5 years	7 (19)	4 (57)	3 (43)	0.021*
	6–10 years	11 (30)	5 (46)	6 (55)	
	11–15 years	12 (32)	6 (50)	6 (50)	
	+ 15 years	7 (19)	3 (43)	4 (57)	
First Aid qualification	No	16 (43)	7 (44)	9 (56)	0.729
	Yes	21 (57)	8 (38)	13 (62)	
BokSmart qualification	No	1 (3)	0 (0)	1 (100)	0.403
	Yes	36 (97)	15 (42)	21 (58)	
School rugby player	No	1 (3)	0 (0)	1 (100)	0.412
	Yes	36 (97)	15 (42)	21 (58)	
Personal concussion experience	Maybe	2 (5)	1 (50)	1 (50)	0.084
	No	9 (24)	2 (22)	7 (78)	
	Yes	26 (70)	12 (46)	14 (54)	
Coaching qualification	None	3 (8)	1 (33)	2 (67)	0.837
	WR Level 1	13 (35)	5 (39)	8 (62)	
	WR Level 2	19 (51)	9 (47)	10 (53)	
	WR Level 3	2 (5)	0 (0)	2 (100)	

^*^Statistically significant *p* < 0.05

## Results

### Participant characteristics

Of the 126 registered rugby coaches contacted on the database, 37 completed the questionnaire resulting in a response rate of 29%. All participants were adult males with an average age of 35.6 years (range 23–60 years), and an average coaching experience of 11.3 years (range 1–30 years; total 418 years). Almost all participants, had a coaching qualification, with most (*n* = 34, 92%) having an advanced coaching qualification namely (World Rugby Levels 1–3), or having a World Rugby Coach Educator License. Almost all participants had completed a BokSmart qualification within the preceding three years; while half of the participants had a first aid certification. All participants had witnessed a concussion, and most had personally sustained a previous concussion injury. All, except one participant, had played rugby at the school level (Table 1).

The Cohen’s effect sizes and odds ratio with 95% confidence intervals for the background variables of participants are presented in Table 2.

**Table 2:**
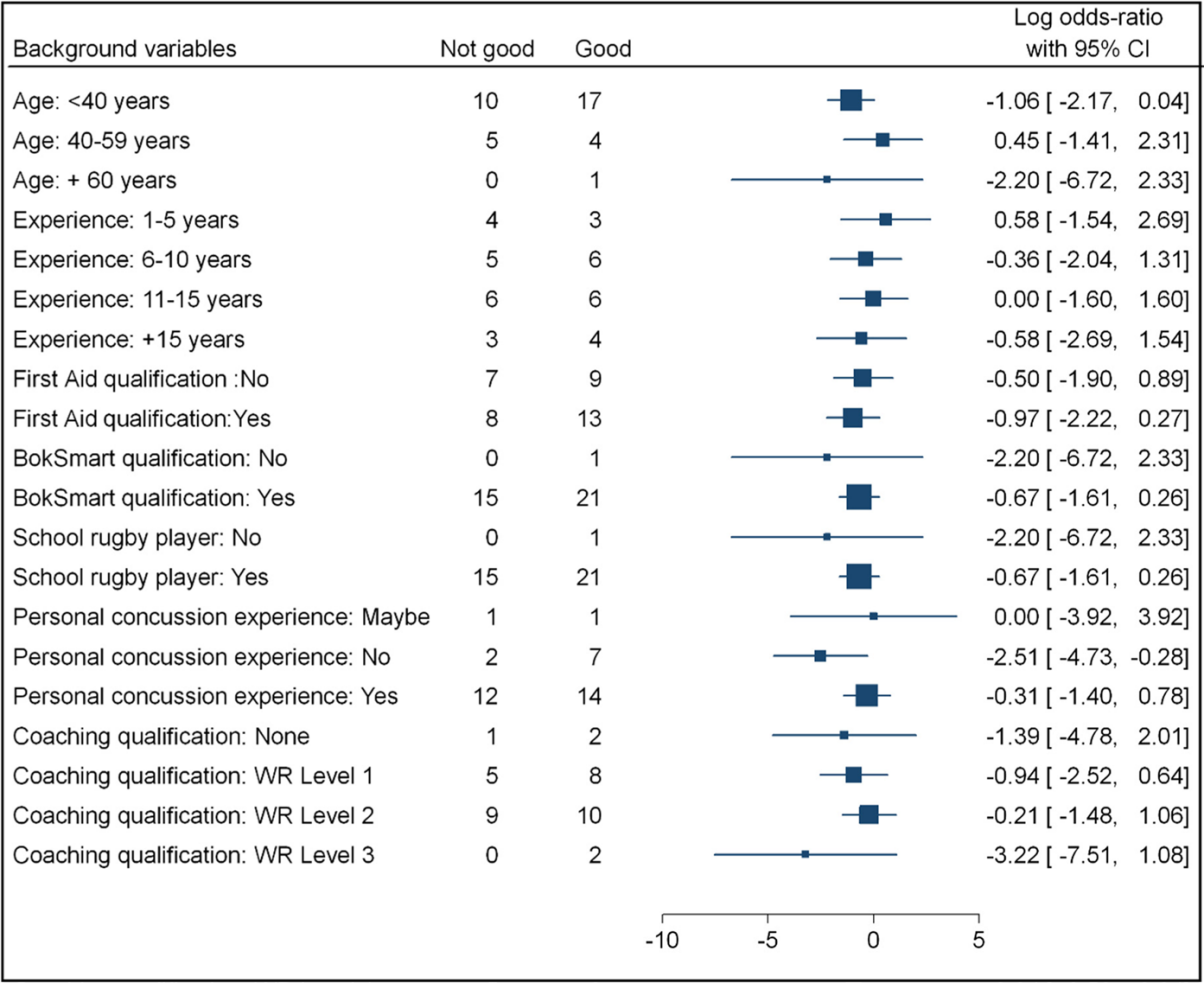
Coach background variables and knowledge categories displayed with confidence interval and effect sizes

### Knowledge

The majority of participants showed good overall knowledge (*n* = 22, 59% vs. *n *= 15, 40%) with participants under 40-years-old, and the only participant over 60 years, showing more ‘good overall knowledge’ than those aged 40–59 years (Tables 1 and 2). Similarly, those with greater coaching experience, a first aid certificate, a recent BokSmart injury prevention programme qualification, or who played rugby at school displayed higher levels of knowledge than participants with less coaching experience, those without a first aid or BokSmart injury prevention programme qualification, or who didn’t play rugby at the high school level. However, only the relationship between good knowledge and rugby coaching experience was found to be statistically significant (*p* < 0.05). Participants achieved higher overall ‘good knowledge’ scores irrespective of having advanced coaching qualifications, however with a large range in the effect size from small to large (-0.21 -3.22), and were more likely to display good overall knowledge if they had not experienced a concussion personally, than if they had.

With regards to specific knowledge regarding concussion in rugby, all but one participant correctly identified a concussion incident as an impairment of brain function after impulsive forces transmitted to the head (*n* = 36; 97%), and all participants identified the appropriate management of a player who had sustained a concussion injury as “removal from the field of play” (*n* = 37; 100%) [55]. Most participants identified that a loss of consciousness was not required when diagnosing a concussion injury and correctly identified some items that may reduce the risk of injury to their players (Table 3). However, half of the participants incorrectly identified “head protection” as an item to reduce the risk of concussion injuries. Some participants correctly identified some risk factors for sustaining a concussion injury, and most participants did not acknowledge that female athletes were at an increased risk of a concussion injury (Table 3). Participants most commonly identified that all paying positions had an ‘equal risk’ of sustaining a concussion injury, followed by those who thought that the loose forwards (*n* = 7;19%); inside backs (*n* = 5;14%); tight five, and front row (*n* = 3; 8%); and lastly outside backs (*n* = 1; 3%) were at greater risk.

**Table 3:** Participants’ responses regarding specific rugby concussion injury knowledge

Knowledge domains	Yes n (%)	Maybe n (%)	No n (%)
**Awareness of World Rugby “Return-to-play” guidelines**	35 (95)	-	2 (5)
**Awareness of school’s “Return-to-play” guidelines**	36 (97)	-	1 (3)
**Loss of consciousness required in concussion**	5 (14)	-	32 (86)
**Awareness of referee’s Blue Card**	34 (92)	3 (8)	1 (3)
Risk factors for concussion			
**Previous concussion**	35 (95)	1(3)	1(3)
**Smaller neck**	6 (16)	7 (19)	24 (32)
**Weaker neck**	29 (78)	5 (14)	3 (8)
Lack of conditioning	34 (92)	3 (8)	0 (0)
** Younger athlete**	21 (57)	4 (11)	12 (32)
** Female athlete**	4 (11)	4 (11)	29 (78)
Items which may reduce the risk of concussion in rugby			
** Mouth guard**	28 (76)	0(0)	9 (24)
Head protection	18 (49)	0 (0)	19 (51)
** Correct tackle technique**	36 (97)	0 (0)	1 (3)
Improved fitness	31 (84)	0 (0)	6 (16)
When should a player return to rugby after sustaining a concussion injury?			
Same day	3 (8)	1 (3)	33 (89)
** Graduated return to play (GRTP)**	25 (68)	0 (0)	12 (32)
** After a doctor has cleared the player**	25 (68)	1 (3)	11 (30)
What are possible long-term effects of concussion?			
**Headaches**	37 (100)	0 (0)	0 (0)
**Eating disorders**	8 (22)	12 (32)	17 (46)
**Depression**	30 (81)	4 (11)	3 (8)
**Substance abuse**	6 (16)	14 (38)	17 (46)
**Memory loss**	36 (97)	1 (3)	0 (0)
Which symptoms may suggest a player has sustained a concussion injury?	Yes n (%)	No n (%)	
** Dizziness**	37 (100)	0 (0)	
** Nausea/vomiting**	37 (100)	0 (0)	
** Confusion**	37 (100)	0 (0)	
** Blurred vision**	36 (97)	1 (3)	
** Emotional changes**	35 (95)	2 (5)	
Coughing	2 (5)	35 (95)	
Choking	2 (5)	35 (95)	
** Involuntary straightening of arms and/or legs following contact to the head**	31 (84)	6 (16)	
** Shivering**	16 (43)	21 (57)	

^*^Bold font = correct answers

#### Symptoms

With respect to symptoms suggestive of a concussion-type injury, all participants responded positively for dizziness, nausea/vomiting, and confusion, and almost all identified blurred vision and emotional changes as symptoms suggestive of a concussion (Table 3). To a lesser extent, the involuntary straightening of arms and/or legs following contact to the head was chosen as a positive symptom of a concussion injury (*n* = 31, 84%). Almost half (43%) of the participants incorrectly identified shivering as a symptom of a potential concussion, and a small number of participants incorrectly identified choking and coughing (*n* = 2, 5%) (Table 3). Most participants identified potential long-term effects of concussion as headaches (*n* = 37, 100), memory loss (*n* = 36, 97%), and depression (*n *= 30, 80%) in descending order, although they did not identify substance abuse or were unsure of this association. A minority of participants (*n* = 8, 22%) associated eating disorders with the long-term effects of concussion (Table 3).

Concerning “Return-to-Play” guidelines following a suspected concussion injury, most participants were aware of both the World Rugby guidelines (*n* = 35, 95%), their school’s guidelines (*n* = 36, 97%)and of the “referee’s blue card” (*n* = 34, 92%), indicative of the referee raising concern of a head injury to a rugby player during a match (Table 2) [56, 57].

### Opinion

In general, the overall six items that addressed coach opinions towards injury prevention and player performance were found to have a mean value of 4.27 (SD ± 0.34), out of a possible 5 maximum points. Player welfare was considered to be more important than player performance (185 vs. 164 respectively) with the item “very important” for player welfare achieving the highest possible score (Table 4). Similarly, the importance of concussion prevention scored very highly (184 out of a possible 185). Most participants felt that recent law changes had improved player safety in rugby, and most participants were satisfied that enough was currently being done to prevent concussion in rugby, although opinions toward rugby becoming ‘too regulated’ were more divided resulting in the greatest standard deviation (Table 4).

**Table 4:** Coaches’ opinions on prevention of injury and player performance

	Not at all n (%)	A little n (%)	Undecided n (%)	Somewhat n (%)	Very n (%)	Total Scores	
						Mean	*SD
Importance of player welfare	0 (0)	0 (0)	0 (0)	0 (0)	37 (100)	5.00	0.00
Importance of concussion prevention	0 (0)	0 (0)	0 (0)	1 (3)	36 (97)	4.97	0.16
Importance of player performance	0 (0)	1 (3)	1 (3)	8 (22)	27 (73)	4.65	0.68
Is rugby becoming too regulated?	9 (24)	12 (32)	3 (8)	10 (27)	3 (8)	2.62	1.34
Do law changes in rugby improve player safety?	0 (0)	0 (0)	7 (19)	19 (51)	11 (30)	4.27	0.80
Is enough being done to prevent concussions?	0 (0)	1 (3)	5 (14)	14 (38)	17 (46)	4.14	0.71
Overall						4.27	0.34

^*^SD *Standard deviation*

Participants identified the doctor as having the greatest responsibility to identify a player who has a concussive injury (184), followed by first aiders (180), the referee (174), the coach (169) and the physiotherapist (168) (Table 4). Other players (139), and parents were considered less responsible (127), and the injured player was considered the least responsible to identify a concussion injury (113), although had the greatest standard deviation of all role players. Similarly, participants also identified the doctor as having the most responsibility to manage a player with a concussive injury (177), followed by the coach (171) and the first aider (163) (Table 4). Other players were considered the least responsible for managing another player’s concussion injury (95), however, opinions differed the most on this item with the greatest standard deviation (SD 1.68).

The provincial sports organisation was considered to have the greatest responsibility for the prevention of concussion injuries (178), followed by the schools’ Director of Sport (177) (Table 4). SA Rugby and coaches followed and were considered to equally share the same level of responsibility (175), before medical personnel (first aider, doctor, physiotherapist), players, Heads of schools, and parents. The Department of Education was considered the least responsible for the prevention of concussion injuries in school children participating in rugby (122) (Table 5).

**Table 5:** Coaches’ opinions towards the responsibility of identification, management and prevention of concussion injuries

Identification of a concussion injury	Not at all n (%)	A little n (%)	Undecided n (%)	Somewhat n (%)	Very n (%)	Total Score	Mean	*SD
Doctor	0 (0)	0 (0)	1 (3)	1 (3)	35 (95)	182	4.92	0.36
First Aider	0 (0)	0 (0)	2 (5)	1 (3)	34 (92)	180	4.86	0.48
Referee	1 (3)	0 (0)	1 (3)	5 (14)	30 (81)	174	4.70	0.78
Coach	1 (3)	1 (3)	0 (0)	9 (24)	26 (34)	169	4.57	0.87
Physiotherapist	1 (3)	2 (5)	2 (5)	3 (8)	29 (78)	168	4.54	1.02
Other players	3 (8)	5 (14)	3 (8)	13 (35)	13 (35)	139	3.76	1.30
Parent	6 (16)	5 (14)	3 (8)	13 (35)	10 (27)	127	3.43	1.44
Injured player	9 (24)	4 (11)	6 (16)	12 (32)	6 (16)	113	3.05	1.45
Management of a concussion injury	Not at all n (%)	A little n (%)	Undecided n (%)	Somewhat n (%)	Very n (%)	Total Score	Mean	*SD
Doctor	0 (0)	0 (0)	2 (5)	4 (11)	31 (84)	177	4.78	0.53
Coach	0 (0)	3 (8)	1 (3)	3 (8)	30 (81)	171	4.62	0.89
First Aider	3 (8)	0 (0)	3 (8)	4 (11)	27 (73)	163	4.41	1.19
Physiotherapist	2 (5)	3 (8)	2 (5)	5 (14)	25 (33)	159	4.30	1.22
Referee	5 (14)	5 (14)	0 (0)	8 (22)	19 (51)	142	3.84	1.52
Parent	6 (16)	5 (14)	3 (8)	9 (24)	14 (38)	131	3.54	1.52
Injured player	7 (19)	3 (8)	7 (19)	8 (22)	12 (32)	126	3.41	1.50
Other players	17 (46)	2 (5)	7 (19)	2 (5)	9 (24)	95	2.57	1.68
Responsible for concussion prevention	Not at all n (%)	A little n (%)	Undecided n (%)	Somewhat n (%)	Very n (%)	Total Score	Mean	*SD
KZN Rugby	0 (0)	0 (0)	0 (0)	7 (19)	30 (81)	178	4.81	0.40
Director of Sport	0 (0)	0 (0)	2 (5)	4 (11)	31 (84)	177	4.78	0.53
Coach	0 (0)	0 (0)	1 (3)	8 (22)	28 (76)	175	4.73	0.51
SA Rugby	0 (0)	0 (0)	1 (3)	8 (22)	28 (76)	175	4.73	0.51
First aider	2 (5)	1 (3)	1 (3)	12 (32)	21 (57)	160	4.32	1.06
Doctor	1 (3)	3 (8)	2 (5)	10 (27)	21 (57)	158	4.27	1.07
Physiotherapist	2 (5)	4 (11)	2 (5)	10 (27)	19 (51)	151	4.08	1.23
Head of School	2 (5)	2 (5)	5 (14)	10 (27)	18 (49)	151	4.08	1.16
Players	2 (5)	2 (5)	6 (16)	11 (30)	16 (43)	148	4.00	1.15
School nurse	3 (8)	5 (14)	7 (19)	9 (24)	13 (35)	135	3.65	1.32
Parents	5 (14)	5 (14)	8 (22)	9 (24)	10 (27)	125	3.38	1.38
Department of Education	7 (19)	5 (14)	5 (14)	10 (27)	10 (27)	122	3.30	1.49
Overall (Responsible for concussion prevention)							4.18	0.55

^*^SD *Standard deviation*

### Barriers

**Fig. 1:**
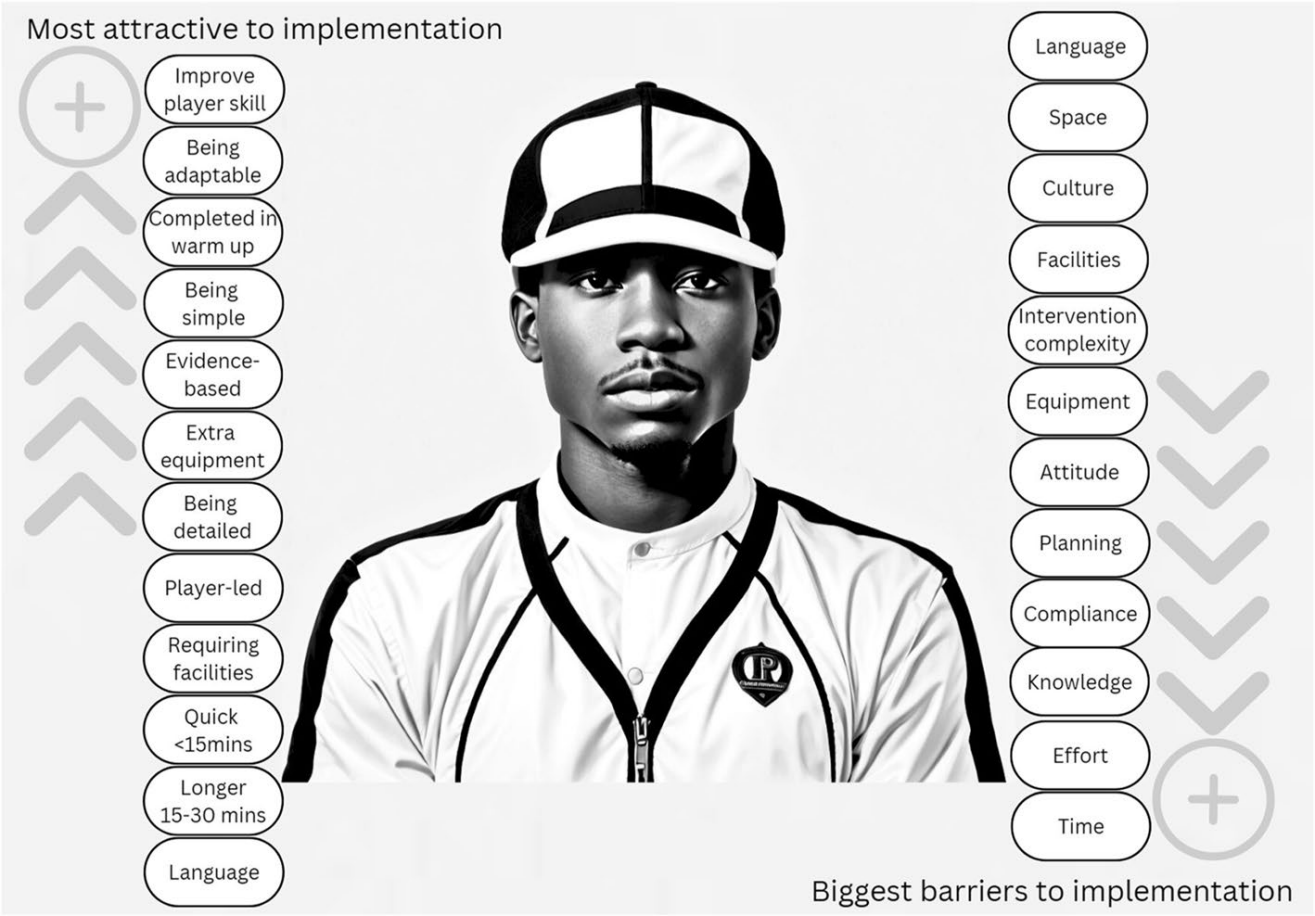
South African coaches’ opinions: attractive characteristics and barriers towards implementing an injury prevention programme (permission obtained)

The biggest barrier reported by participants to implementing an injury prevention programme was time (total score 138, SD ± 1.59, mean 3.73) followed closely by effort (130, ± 1.56, 3.51), compliance (127, ± 1.68, 3.43), knowledge (127, ± 1.68, 3.43), planning (126, ± 1.69, 3.41), attitude (124, ± 1.69, 3.35), equipment (117, ± 1.73, 3.30) and intervention complexity (116, ± 1.46, 3.14) (Fig. 1). The lowest reported barriers to implementation were facilities (total score 109, SD ± 1.82, 2.95), culture (105, ± 1.61, 2.84), space (86, ± 1.56, 2.32), and language (76, ± 1.49, 2.05) (Fig. 1).

### Attractive characteristics

The most attractive characteristic of an injury prevention programme was to improve player skill (total score 173, SD ± 0.75, mean 4.68), followed by being adaptable (171, ± 0.86, 4.62), and being completed in the warm-up at the start of a training session (167, ± 0.93, 4.51). Interestingly, injury prevention programmes that were not included in the warm-up were the least attractive (total score 63, SD ± 1.24, mean 1.70), with more than two-thirds of participants reporting ‘not at all’ attractive (*n* = 26) (Fig. 1).

Less appealing characteristics, in descending order, were being simple (160, ± 4.32, 0.94), being evidence-based (158, ± 1.26, 4.27), requiring extra equipment (157, ± 1.28, 4.24), being detailed (149, ± 1.44, 4.03) being player led (134, ± 1.36, 3.62) and requiring facilities (113, ± 1.47, 3.05). A session considered to be quick, of less than 15 min in duration, was only slightly more attractive than a longer session of 15–30 min (123, ± 1.49, 3.32 vs 117, ± 1.61, 3.16) (Fig. 1).

More than half of the participants reported doing some form of baseline testing (Sports Concussion Assessment Tool 5, *n* = 5, 14%; clinical testing, *n* = 11, 30%; HeadSmart, *n* = 1, 3%; none, *n* = 20, 54%) prior to their players sustaining a concussion, and most participants used the Concussion Recognition Tool (*n* = 25, 68%) following a head impact to their players. Participants identified the frequency of coach-player discussions regarding concussion injuries was similar to that between coaches and other coaches (126, 68% vs. 120, 65% respectively).

## Discussion

### Knowledge

The main finding of this study was the identification of the knowledge and opinions regarding concussion injuries of a cohort of High School Rugby coaches in one province of South Africa. Previous concerns about the dissemination of concussion information and guidelines to amateur and community players in Australia are similar in this study [58, 59]. In this study, although all coaches had completed the BokSmart training prior to testing, only half had a good overall general knowledge of concussion injuries, with greater rugby coaching experience being the only significant differentiating factor. This finding does however support repeated coach training and re-accreditation where knowledge may be retained through greater exposure, a technique seen in other adult education approaches in the US [60]. Notably, this is the chosen method of the biennial SA Rugby BokSmart Rugby Safety accreditation programme for coaches and referees, supplemented with online resources since 2009 [61, 62]. Further, the BokSmart Programme aims to reduce the burden of all injuries, especially catastrophic injuries, with a specific emphasis placed on education on concussion [61].

With regard to specific concussion knowledge, correct tackle technique was identified as being associated with reducing the risk of head injury and is recommended by World Rugby to be an appropriate technical element that high school rugby coaches should be addressing during training sessions and matches [63]. Another element that was appropriately recognized and pertinent to this cohort, was that of physical and cardiorespiratory fitness preparation of players. Research from the US, Australia, Canada, Ireland and the UK has demonstrated that lower fitness levels have been associated with an increased risk of injury in sub-elite athletes, and lower neck strength with in an increase in concussion incidence [64–67].

Interestingly, half of the participants identified rugby headgear as an item to reduce the risk of concussion, similar to previous research findings [42]. However, headgear may have no effect [68, 69], or may result in a change in behaviour of athletes when wearing headgear, referred to as the risk-compensation phenomenon, thus increasing the chances of concussion, especially in contact sports [38, 69, 70]. This misconception should be addressed as coaches are an important source of information for players and parents, especially at school levels.

Another item that divided the cohort almost equally, was that of shivering as a symptom of concussion. This sign to raise the body’s temperature should be distinguished from the myoclonic or tonic–clonic motor jerks occasionally occurring immediately after a head trauma/injury [71]. Although this symptom is reported as uncommon in the literature [71], it is addressed in the BokSmart Concussion Guidance and World Rugby’s Player Welfare resources [72, 73], and listed on the Concussion Recognition Tool (CRT6) designed for non-medically trained individuals (coaches) [74], and on the Sports Concussion Assessment Tools (SCAT6, or Child SCAT6), designed for medical personnel [75, 76].

It may be debated as to whether *all* concussion information is pertinent to coaches, especially with regard to the possible long-term symptoms or effects, as their role may be considered more important in the prevention and identification of concussion injuries. However, the authors would argue that an understanding of both the acute and chronic signs and symptoms of a concussive injury is critical and may not be easily identifiable. Freely available and regularly updated tools, like the Concussion Recognition Tool 6 (CRT6), are designed to provide guidance in improving the health and well-being of athletes, and should be routinely used [77].

Whilst some of the results of specific concussion knowledge differed between coaches, critical components of BokSmart and WR concussion initiatives were consistently observed in the ‘Recognise and Remove’ and ‘Return-to-play’ guidelines for a player with a suspected concussion injury [72, 78]. This generally widespread understanding is supported by the opinion scores of concussion prevention being very important to high school rugby coaches. However, although coaches appeared to have the appropriate knowledge, a small number still reported that they would let a player return to play on the same day of sustaining a concussion injury, supporting recent findings in community club rugby stakeholders which are contrary to player welfare guidelines [38, 42]. Further, one-third of coaches did not advocate for players returning to rugby after a graduated return to play (GRTP) or after a doctor had cleared the player, which opposes both World Rugby and BokSmart protocols [72, 79].

With regard to specific concussion knowledge, the risk to female athletes and younger athletes was underreported by this cohort [80–82]. Although not a consistent finding in adult Women’s rugby [83, 84], and the limited research in female adolescent rugby players [82], a significantly greater risk of concussion injuries, and longer recovery times, has been identified in female high school athletes in other contact sports like baseball, basketball and soccer [80, 85]. With the rapid growth in Women’s rugby globally, with almost a third more players now registered than in 2017, and women accounting for more than 25% of the overall playing population, further research in this area is recommended [80, 86].

Additionally, this study identified the opinions of high school rugby coaches towards injury and concussion prevention. It is encouraging that player welfare is considered the highest priority, and concussion prevention rated higher than player performance, especially in a competitive high school rugby playing community that includes professional coaches, sports scientists, and high-performance programmes and centres [87, 88]. Fittingly, improving player skill was the most appealing feature of an injury prevention programme, reinforcing this as a priority area for coaches. Coaches valued time and efficiency as highly attractive features of an injury prevention programme that is completed in the warm-up, coinciding with the most significant reported barriers to implementing an injury prevention programme being the required time and effort.

With respect to opinions regarding responsibility, coaches considered doctors to have the highest responsibility for both identifying and managing concussion injuries in players. While first aiders are required to be at all normal school rugby matches, but not at training sessions [89], most community-based teams did not employ qualified medical support staff [90], Doctors were the least likely members to be at community rugby matches and most likely to be absent at high school rugby training sessions, where a more controlled playing environment has previously been described, with a lower risk of a concussion injury [89, 91]. The coaches in this study regarded the first aider as having the most responsibility for the prevention of concussions, which is encouraging as many injury prevention initiatives provide and mandates rugby-specific first aid training for example; The BokSmart Rugby Medic programme, England Rugby Emergency First aid in Rugby Union (EFARU) and WR First aid in Rugby (FAIR) courses [92–94].

The provincial sport’s governing body (KZNRU) was considered to have the most responsibility for preventing concussions, followed by the school sports administration, which is similar to previous international findings [95]. While coaches acknowledged a certain degree of responsibility toward identifying, managing, and preventing concussion injuries, they conveyed much less responsibility for the players in this regard. As key stakeholders for the BokSmart programme, this is an avenue that should be explored in greater detail in further studies. In contrast to other countries where concussion is seen as a Public Health issue [96–98], the Government Department of Education was reported to be the least responsible for preventing concussions in school sports.

The results of this study provide valuable information and insights into coaches' knowledge, opinions a behaviours with real-world relevance. The insights may impact future policy and practice for the design of future concussion education but also for the development of concussion prevention programmes which are dependent on coach adherence.

Future research should include longitudinal studies to evaluate changes in knowledge and attitudes over time, and gaps or trends in the content of concussion education initiatives. Alternate methods of adult learning may also be incorporated as these may be considered more appropriate to knowledge retention than taught sessions, and warrant further exploration, especially with the rapid advancements in digital education and simulation-based education. Future educational and knowledge transfer strategies could be tailored to individual needs based on a pre-course assessment, thus ensuring the most appropriate use of time and resources to ensure the appropriate knowledge transfer to each individual.

#### Limitations

Purposive samples are prone to researcher bias [51], however, the participants included were anonymous to the researcher which reduces this bias. The answers were self-reported and open to bias and socially acceptable answers, especially with the considerable amount of attention surrounding concussions in the mainstream media. The implications for bias due to phrasing should be considered in the development of future research tools [99]. The response rate for the questionnaire was low but acceptable (29%), even after follow-up invitations to participate in the study, and are open to manipulation [99]. The response rate was considered ‘acceptable’ when compared to a previous study using a questionnaire research tool via email in coaches (18%) [100]. Increased participation could have provided more information on the current research aims and reduced the homogeneity of the sample, as all participants were adult males. Thus, the opinions, knowledge and behaviour of the vast majority of KZN High School rugby coaches could not be included in this analysis, and are not representative, especially coaches of different genders. Non-response bias highlights that the opinions, knowledge and behaviour of non-respondents may differ significantly on different factors from those who did complete the questionnaire [99].

Payment for participation was not an option due to the limited resources of this study, however, this was considered. Extending the reach to participants may have increased participation and future designs should include the use of social media and social media ‘influencers’ to recruit a greater number of participants.

Although a pilot study was conducted, the research tool was not assessed for reliability. The Likert scale may have reduced the effect of central tendency bias [101]. The official communication from the High School Rugby Association is in the English language, however the researchers acknowledge the questionnaire was only provided in English in South Africa, a country with 11 official languages. Coaches with limited internet access may have been excluded unknowingly from this study, as the questionnaires were distributed online. Similarly, there may be high school coaches in KZN who are not registered with the KZNRU. Lastly, the study was conducted during the COVID-19 global shutdown of all sports, although this was unlikely to influence knowledge or opinions.

## Conclusion

Our results assist in a greater understanding of the knowledge and opinions of key stakeholders in Rugby Union, namely high school rugby coaches, regarding concussion prevention, responsibility for the identification, management, and prevention of concussion, and barriers and preferences to implementing a concussion prevention programme. An injury prevention programme that would improve player skill whilst reducing the risk of injury, is adaptable and not take much time or effort to complete, is the preference of the coaches in this study. These findings support the coach-focused education strategy of the biennial accreditation required by SA Rugby’s BokSmart programme while also identifying knowledge gaps and providing insight into specific improvements in concussion education initiatives in rugby.

### Supplementary Information

Supplementary Material 1.

## Data Availability

No datasets were generated or analysed during the current study.

## Abbreviations

CISG: Concussion in Sport Group
CRT: Concussion Recognition Tool
IBM: International Business Machines
GRTP: Graduated Return to Play
KZN: KwaZulu-Natal
NMT: Neuromuscular training
SCAT: Sports Concussion Assessment Tool
SD: Standard deviation
UKZN: University of KwaZulu Natal
WR: World Rugby
