# Supplementary material for: High school rugby coaches’ knowledge and opinions of concussion in KwaZulu Natal province in South Africa: an ecological cross-sectional study
Source: BMC Sports Sci Med Rehabil. 2024 Jun 24;16:139. doi: 10.1186/s13102-024-00930-5 (PMC11194973; doi:10.1186/s13102-024-00930-5)
Supplement: Supplementary file 1 — Supplementary Material 1. [file 13102_2024_930_MOESM1_ESM.docx]

Appendix A.

Coach Questionnaire

The purpose of this questionnaire is to gain insight into the:

Knowledge and opinions of South African rugby coaches around the identification and management of concussion in training and matches.

Barriers, concerns or strategies to implementing an injury prevention programme in rugby in South Africa.

Personal information will be treated in the strictest confidence and will only be available to the principal investigator. A study number will be allocated to each participant when the data are being captured assuring anonymity and confidentiality.

This information from this questionnaire will prove invaluable in our understanding and development of concussion prevention strategies that will allow improved player welfare in the game of rugby. Date. yyyy/mm/dd

| Coach Information | | | | | | |
| --- | --- | --- | --- | --- | --- | --- |
| Surname |  | | | | | |
| First name |  | | | | | |
| Date of birth |  | | | | | |
| School name |  | | | | | |
| What team(s) are you coaching this year? |  | | | | | |
| Coaching Qualification | WR Level 1 | WR Level 2 | | | | WR Level 3 |
| Other/none: | | | | | | |
| Coaching Experience | months | | | years | | |
| First Aid Qualification | yes | | | no | | |
| BokSmart Accreditation | yes | | | no | | |
| Most recent BokSmart Accreditation | (year) | | | (Union) | | |
| Did you play rugby at school? | yes | | | no | | |
| Have you personally suffered a concussion? | Yes | | no | | unsure | |
| Have you seen a player who has been concussed? | Yes | | no | | unsure | |

| Question 1: Do you know what concussion is? (Please mark one answer) | | |
| --- | --- | --- |
| Condition in the body that doesn’t allow sugar to be regulated in the blood. | Impairment of brain function after impulsive forces transmitted to the head. | Serious infectious disease of the lungs caused by bacteria. |

| Question 2: Please rate the following questions on a scale of 1-10 where 1 is the lowest value and 10 is the highest value: |
| --- |

2.1 How important is it to you, to prevent injury to your players?

| 1 | 2 | 3 | 4 | 5 | 6 | 7 | 8 | 9 | 10 |
| --- | --- | --- | --- | --- | --- | --- | --- | --- | --- |

2.2 How important is player performance to you?

| 1 | 2 | 3 | 4 | 5 | 6 | 7 | 8 | 9 | 10 |
| --- | --- | --- | --- | --- | --- | --- | --- | --- | --- |

2.3 How important is prevention of injury to opposition players to you?

| 1 | 2 | 3 | 4 | 5 | 6 | 7 | 8 | 9 | 10 |
| --- | --- | --- | --- | --- | --- | --- | --- | --- | --- |

2.4 Does better injury prevention improve player performance?

| 1 | 2 | 3 | 4 | 5 | 6 | 7 | 8 | 9 | 10 |
| --- | --- | --- | --- | --- | --- | --- | --- | --- | --- |

2.5 Does better player performance improve injury prevention?

| 1 | 2 | 3 | 4 | 5 | 6 | 7 | 8 | 9 | 10 |
| --- | --- | --- | --- | --- | --- | --- | --- | --- | --- |

| Question 3: Do you think that the game of rugby is becoming too regulated? | | | | |
| --- | --- | --- | --- | --- |
| Not at all | A little | Undecided | Somewhat | Very |
| 1 | 2 | 3 | 4 | 5 |

| Question 4: How important is player performance to you? | | | | |
| --- | --- | --- | --- | --- |
| Not at all | A little | Undecided | Somewhat | Very |
| 1 | 2 | 3 | 4 | 5 |

| Question 5: How important is player welfare to you? | | | | |
| --- | --- | --- | --- | --- |
| Not at all | A little | Undecided | Somewhat | Very |
| 1 | 2 | 3 | 4 | 5 |

| Question 6: Is preventing concussion important to you? | | | | |
| --- | --- | --- | --- | --- |
| Not all important | Not too important | Undecided | Somewhat | Very important |
| 1 | 2 | 3 | 4 | 5 |

| Question 7: Do you feel that you have the necessary knowledge and skills to identify concussion effectively? | |
| --- | --- |
| YES | NO |

| Question 8: Which of these are risk factors for concussion? | | | |
| --- | --- | --- | --- |
|  | Yes | No | Unsure |
| Previous concussion |  |  |  |
| Smaller neck |  |  |  |
| Weaker neck |  |  |  |
| Lack of conditioning |  |  |  |
| Younger athletes (children) |  |  |  |
| Female athletes |  |  |  |

| Question 9: Can the following reduce the risk of concussion? | | | |
| --- | --- | --- | --- |
|  | Yes | No | Unsure |
| Wearing a gum guard |  |  |  |
| Wearing head protection |  |  |  |
| Correct tackle technique |  |  |  |
| Improved fitness |  |  |  |
| Pre-activity exercises in the warm-up |  |  |  |

| Question 10: Which positions are at greatest risk of sustaining a concussion? | | |
| --- | --- | --- |
| Inside backs | Outside backs | Tight five |
| Loose forwards | Front row | All have the same risk of being injured |

| Question 11: Whose responsibility is it to IDENTIFY athletes who are concussed? | | | | | |
| --- | --- | --- | --- | --- | --- |
|  | Not at all | A little | Undecided | Somewhat | Definitely |
| Referee | 1 | 2 | 3 | 4 | 5 |
| Coach | 1 | 2 | 3 | 4 | 5 |
| First Aider / Paramedic | 1 | 2 | 3 | 4 | 5 |
| Doctor | 1 | 2 | 3 | 4 | 5 |
| Physiotherapist | 1 | 2 | 3 | 4 | 5 |
| Parent | 1 | 2 | 3 | 4 | 5 |
| Other players | 1 | 2 | 3 | 4 | 5 |
| The injured player | 1 | 2 | 3 | 4 | 5 |

| Question 12: Whose responsibility is it to MANAGE athletes who are concussed? | | | | | |
| --- | --- | --- | --- | --- | --- |
|  | Not at all | A little | Undecided | Somewhat | Definitely |
| Referee | 1 | 2 | 3 | 4 | 5 |
| Coach | 1 | 2 | 3 | 4 | 5 |
| First Aider / Paramedic | 1 | 2 | 3 | 4 | 5 |
| Doctor | 1 | 2 | 3 | 4 | 5 |
| Physiotherapist | 1 | 2 | 3 | 4 | 5 |
| Parent | 1 | 2 | 3 | 4 | 5 |
| Other players | 1 | 2 | 3 | 4 | 5 |
| The injured player | 1 | 2 | 3 | 4 | 5 |

| Question 13: Which of these symptoms may suggest a concussion?  (Please choose more than one answer if applicable) | | | |
| --- | --- | --- | --- |
|  | Yes | No | Unsure |
| Dizzy |  |  |  |
| Nausea/vomiting |  |  |  |
| Blurred vision |  |  |  |
| Confusion |  |  |  |
| Emotional changes (e.g. crying, aggression) |  |  |  |
| Coughing |  |  |  |
| Choking |  |  |  |
| Shivering |  |  |  |
| Involuntary straightening of arms or legs after contact to the head |  |  |  |

| Question 14: What should be done if a player is suspected of having a concussion?  (Please choose one answer) | | | |
| --- | --- | --- | --- |
|  | Yes | No | Unsure |
| Monitor for 5-10 minutes before making a decision |  |  |  |
| Let him run it off |  |  |  |
| Removal from training/match |  |  |  |
| Apply ice for 10-20 mins before returning to play |  |  |  |

| Question 15: With regards to injury prevention exercises programmes, are you familiar with…  (Please choose more than one answer if applicable) | | |
| --- | --- | --- |
|  | Yes | No |
| Safe Six exercises |  |  |
| FIFA 11 + exercises |  |  |
| Activate exercises |  |  |
| Other: Please list… | | |

| Question 16: Do you use any of these injury prevention exercises in your warmups?  (Please choose more than one answer if applicable) | | |
| --- | --- | --- |
|  | Yes | No |
| Safe Six exercises |  |  |
| FIFA 11 + exercises |  |  |
| Activate exercises |  |  |
| Other: Please list… | | |

| Question 17: How did you learn about these injury prevention exercises  (Please choose more than one answer if applicable) | | | | | |
| --- | --- | --- | --- | --- | --- |
| I don’t use injury prevention exercises | Coach | SA Rugby | Provincial Union | email | Social media |
| Other: Please list… | | | | | |

| Question 18: When should a player return to rugby following a concussion? | | | |
| --- | --- | --- | --- |
|  | Yes | No | Unsure |
| When the player is symptom-free on the same day |  |  |  |
| After following a graduated return-to-play protocol |  |  |  |
| After a doctor has cleared the player |  |  |  |
| When the player is symptom free, after following a graduated return-to-play protocol and after being cleared by a doctor |  |  |  |

| Question 19: What are possible long-term symptoms of concussion?  (Please provide an answer for each item) | | | |
| --- | --- | --- | --- |
|  | Yes | No | Unsure |
| Severe headaches |  |  |  |
| Eating disorders |  |  |  |
| Depression |  |  |  |
| Substance/alcohol abuse |  |  |  |
| Memory problems |  |  |  |

| Question 20: Are you aware of the World Rugby return to play guidelines after concussion? | |
| --- | --- |
| Yes | No |

| Question 21: How often do you discuss injuries with your players? | | | | |
| --- | --- | --- | --- | --- |
| Not at all | Rarely | Undecided | Occasionally | Very |
| 1 | 2 | 3 | 4 | 5 |

| Question 22: How often do you discuss concussion with your players? | | | | |
| --- | --- | --- | --- | --- |
| Not at all | Rarely | Undecided | Occasionally | Very |
| 1 | 2 | 3 | 4 | 5 |

| Question 23: Does your school/club” have concussion protocols that you are aware of? | |
| --- | --- |
| Yes | No |

| Question 24: What are the major barriers to implementing an injury prevention programme? | | | | | |
| --- | --- | --- | --- | --- | --- |
|  | Not at all | A little | Undecided | Somewhat | Definitely |
| Attitude | 1 | 2 | 3 | 4 | 5 |
| Time | 1 | 2 | 3 | 4 | 5 |
| Culture | 1 | 2 | 3 | 4 | 5 |
| Language | 1 | 2 | 3 | 4 | 5 |
| Space | 1 | 2 | 3 | 4 | 5 |
| Facilities | 1 | 2 | 3 | 4 | 5 |
| Equipment | 1 | 2 | 3 | 4 | 5 |
| Effort | 1 | 2 | 3 | 4 | 5 |
| Knowledge | 1 | 2 | 3 | 4 | 5 |
| Compliancy | 1 | 2 | 3 | 4 | 5 |
| Planning | 1 | 2 | 3 | 4 | 5 |
| Intervention complexity | 1 | 2 | 3 | 4 | 5 |

| Question 25: Do you feel you have sufficient knowledge regarding concussion? | |
| --- | --- |
| Yes | No |

| Question 26: Is a loss of consciousness (blackout) required when suspecting a concussion? | |
| --- | --- |
| Yes | No |

| Question 27: What items of an injury prevention programme would be attractive to you as a coach? | | | | | |
| --- | --- | --- | --- | --- | --- |
|  | Not at all | A little | Undecided | Somewhat | Definitely |
| Quick <15mins | 1 | 2 | 3 | 4 | 5 |
| Longer 15-30mins | 1 | 2 | 3 | 4 | 5 |
| Detailed | 1 | 2 | 3 | 4 | 5 |
| Simple | 1 | 2 | 3 | 4 | 5 |
| Included in a warm-up | 1 | 2 | 3 | 4 | 5 |
| Not included in warm up | 1 | 2 | 3 | 4 | 5 |
| No Equipment | 1 | 2 | 3 | 4 | 5 |
| Player led | 1 | 2 | 3 | 4 | 5 |
| Facilities | 1 | 2 | 3 | 4 | 5 |
| Effective/ Evidence based | 1 | 2 | 3 | 4 | 5 |
| Improve player skill | 1 | 2 | 3 | 4 | 5 |
| Adaptable | 1 | 2 | 3 | 4 | 5 |

| Question 28: Are there other items of an injury prevention programme that are not listed that would be attractive to you as a coach? |
| --- |
| Other |

| Question 29: Do you understand what the referee’s blue card means? | | |
| --- | --- | --- |
| Yes | No | Unsure |

| Question 30: Have recent protocols by SA Rugby and World Rugby improved player safety? | | | | |
| --- | --- | --- | --- | --- |
| Not at all | A little | Undecided | Somewhat | Very |
| 1 | 2 | 3 | 4 | 5 |

| Question 31: Do you use the Concussion Recognition Tool? | |
| --- | --- |
| Yes | No |

| Question 32: Do the players in your team do baseline testing for concussion? | | | |
| --- | --- | --- | --- |
| SCAT5 | Clinical testing | Other | No |

| Question 33: Do the players in your team do computerized baseline testing for concussion? | | | |
| --- | --- | --- | --- |
| ImPACT | CogState Sport (CogSport) | Other | No |

| Question 34: Do the players in your team do testing AFTER concussion? | | | | |
| --- | --- | --- | --- | --- |
| SCAT5 | ImPACT | CogState Sport (CogSport) | Other: | No |

| Question 35: Do you think enough is being done to prevent concussion? | | | | |
| --- | --- | --- | --- | --- |
| Not at all | A little | Undecided | Somewhat | Very |
| 1 | 2 | 3 | 4 | 5 |

| Question 36: Have recent law changes to the game of rugby improved player safety? | | | | |
| --- | --- | --- | --- | --- |
| Not at all | A little | Undecided | Somewhat | Very |
| 1 | 2 | 3 | 4 | 5 |

| Question 37: Who should be responsible for driving concussion prevention measures at your school? | | | | | |
| --- | --- | --- | --- | --- | --- |
|  | Not at all | A little | Undecided | Somewhat | Definitely |
| Coach | 1 | 2 | 3 | 4 | 5 |
| Director of Sport | 1 | 2 | 3 | 4 | 5 |
| First Aiders/ Paramedics | 1 | 2 | 3 | 4 | 5 |
| Physiotherapists | 1 | 2 | 3 | 4 | 5 |
| Doctors | 1 | 2 | 3 | 4 | 5 |
| Players | 1 | 2 | 3 | 4 | 5 |
| Department of Education | 1 | 2 | 3 | 4 | 5 |
| SA Rugby | 1 | 2 | 3 | 4 | 5 |
| KZN Rugby | 1 | 2 | 3 | 4 | 5 |
| Headmaster | 1 | 2 | 3 | 4 | 5 |
| Parents | 1 | 2 | 3 | 4 | 5 |
| School nurse | 1 | 2 | 3 | 4 | 5 |
